# Stable Two-Sided Satisfied Matching for Hospitals and Patients Based on the Disappointment Theory

**DOI:** 10.1007/s44196-022-00138-w

**Published:** 2022-09-14

**Authors:** Xiaojia Wang, Rong Wang, Shanshan Zhang, Junhang Liu, Li Jiang

**Affiliations:** 1 Institute of Artificial Intelligence and Data Science, School of Management, Hefei University of Technology, No. 193, Tunxi Road, Mailbox 270, Hefei, 230009 Anhui China; 2 Department of Clinical Teaching, The First Affiliated Hospital of Anhui University of Chinese Medicine, Hefei, 230031 Anhui China; 3 The National Chinese Medicine Clinical Research Base-Key Disease of Diabetes Mellitus Study, Hefei, 23003 Anhui China

**Keywords:** Stable TSM, Disappointment theory, TOPSIS, Uncertain preference order, Fairness weight

## Abstract

With the global spread of COVID-19 and the shortage of medical resources, the key to improve the quality of medical services is to solve the problem of hospital–patient matching. This paper constructs a two-sided matching (TSM) model based on the psychological perceptions of hospitals and patients to realize effective matching that maximizes the satisfaction of hospitals and patients. First, we determine the influencing factors of mutual choice between hospitals and patients through investigation and literature and establish a TSM evaluation system to obtain the preference order of hospitals and patients. Then, using disappointment theory, the preference order value is transformed into preference utility, and the preference utility of hospitals and patients is transformed into the perceived utility of hospital and patient satisfaction. Finally, under the constraint of stable matching, a multiobjective optimization model of TSM is established with the goal of maximizing the sum of the perceived utility of hospitals and patients. The optimal TSM results are obtained by solving the model, and an example is given to verify the practicability and effectiveness of the model. The results show that the stable bilateral satisfaction matching model considering the psychological factors of both sides can fully meet the expectations of hospitals and patients and has certain practical value.

## Introduction

The sudden outbreak of COVID-19 has further stressed already scarce medical resources. Even though Wuhan has abundant medical resources as a leading city in medical radiation, the sudden collapse caused by COVID-19 caused the collapse of the medical system, which could not effectively treat patients. Therefore, effectively configuring the medical resources to meet the needs of hospitals and patients during COVID-19 is worthy of deeper reflection.

The present study was conducted by a local health management organization. The senior management of the organization wants to find a way to meet the needs of hospital patients to the greatest extent through a reasonable allocation of limited medical resources to alleviate the contradiction between the supply and demand of medical resources. For the sake of confidentiality, the virtual name “Medical Research Institute” (MRI) is used to refer to the organization.

MRI is a professional and authoritative research institution in the field of medical and health research and development. It actively engages in academic exchange and cooperation in the medical and health realms in China and abroad; promotes the development of medical and health undertakings; conducts research on public health theory, health economics theory, health policy and health development and reform issues; and transforms research results into policy suggestions, which are provided to the Chinese government and its relevant departments for decision-making reference.

Therefore, in the context of the COVID-19 pandemic, MRI is interested in exploring the matching of medical resources and determining how to maximize the satisfaction of both sides. MRI has investigated and studied factors that affect the mutual selection of hospitals and patients, and this paper mainly uses existing information and data for collation and analysis and establishes a matching model to achieve the maximum satisfaction of both sides.

Medical service supply and demand matching essentially belongs to the category of TSM (two-sided matching) decision-making. Early research on the TSM problem originated from the research on marriage matching between men and women and the university admission problem described by American scholars Gale and Shapley [1]. In the practical TSM problem, forming a reasonable and effective TSM result is the common requirement of all parties involved in matching. The TSM problem based on preference order has been deeply studied by scholars. Foreign scholars such as Gale and Shapley [1] and Roth [12] and domestic scholars such as Li Mingyang [9] and Liang Haiming [27] studied the TSM problem based on preference order. At present, in-depth study of the TSM model based on the preference order is still needed mainly based on the following considerations:

1. The current research focuses on marriage matching between men and women [2], matching of student enrollment [3], matching of employees and job roles [4], and matching of transactions between buyers and sellers [5]. Few researchers have studied the hospital−patient resource matching problem.
2. Most studies use deferral algorithms (including the Gale–Shapley algorithm, the hospital-resident algorithm and their improved and extended algorithms). These algorithms are usually given from the perspective of stable TSM and seldom consider the overall satisfaction and fair treatment of both parties.
3. The existing research on this issue is still in its infancy and lacks systematic analysis and discussion. At present, some studies explore preference order-based TSM methods considering the satisfaction of both parties [5, 26]. However, the existing studies have not systematically characterized the satisfaction function of both parties by preference order and do not consider the stability of TSM results.
4. Both subjects’ satisfaction with the matching results is closely related to their psychological and behavioral factors, but there is no research on this issue. Specifically, there will be a disappointment–elation psychological perception of the matching results among the two parties of the matching decision makers, and this psychological perception is closely related to the satisfaction of the two parties. If the satisfaction of the two parties in TSM is carefully described, it is necessary to introduce behavioral decision theory into the study of bilateral matching.

An in-depth study of the two-sided satisfactory matching method based on preference order solves the problem of TSM between hospitals and patients. It is necessary to promote TSM research and lay a foundation for hospital and patient matching research.

As our main contribution, we propose a multiobjective optimization model with the objective of maximizing the sum of the perceived utilities of hospitals and patients and provide a reasonable and effective medical service supply and demand matching proposal. This can not only meet the different needs of patients but also further reduce social medical costs, improve the efficiency of the medical industry, improve the satisfaction of both hospitals and patients, and improve the competitiveness of the entire medical service industry. In addition, based on the disappointment theory of behavioral decision theory, stable TSM is used to determine a reasonable matching result based on the matching preference information of both parties and to meet the preferences and requirements of both parties as much as possible, and a model considering the psychological behavior of both parties is proposed. The model uses the perceptual effect of the subject’s psychological perception of elation and disappointment to measure the subject’s satisfaction with the TSM result.

The remainder of this paper is organized as follows. In Sect. 2, we review the related literature. In Sect. 3, we establish two comprehensive evaluation systems and describe the perceived utilities of hospitals and patients. In Sect. 4, an one-to-one TSM multiobjective optimization model is constructed based on the preference order. Based on the weighted sum method of the membership function, the multiobjective optimization model is transformed into a single objective optimization model, and the optimal TSM result is obtained by solving the model. In Sect. 5, we present a hypothetical case study of hospital–patient matching (HPM). We summarize and conclude this study in Sect.6.

The following is an introduction to some of our theoretical foundations to illustrate disappointment theory and TSM used in modeling.

### Disappointment Theory

According to disappointment theory [6], the loss of a certain amount of property brings more disappointment than the elation experienced from the same income; that is, for the same amount of income and loss, people perceive the loss more. For subjects involved in TSM, disappointment aversion psychology is also at play. If we want to describe this concept, we need to introduce the disappointment elimination function.

Bell [7] proposed disappointment theory in 1985 to examine the deviation between people’s actual behavior and expected utility theory. According to disappointment theory, disappointment refers to an emotion that people experience when they experience multiple expected results before an event but obtain poor results after the event. The basic idea of disappointment theory is the following: people will compare their present situation with their expectations. When the current situation falls short of their expectations, people will feel disappointed; otherwise, they will feel happy. Loomes and Sugden [28] considered disappointment and elation to be key factors involved in making rational choices. Delquié and Cillo [29] show that an individuals’ disappointment or satisfaction with a result is unrelated to the expected utility because any result can only be expected based on its probability to some extent, and it is difficult to choose an index to accurately predict a result. In TSM, the subject has psychological perceptions of disappointment and elation for matching results. When one is happy to match with the other subject of the current matching object, one will also be disappointed if one fails to match with an opposite subject that is better than the current matching object. To describe the rational behavior of both parties, we can use the disappointment theory of behavioral decision theory for reference.

### Two‑Sided Matching Problem

The work of American scholars Gale and Shapley [1] on marriage matching and college admission constitute the origins of this field. Since these works, Professor Roth [30] of the University of Pittsburgh and other scholars have conducted in-depth research on TSM and clearly defined the concept of TSM. Zhang Zhenhua [31], Chen Xihe and Fan Zhiping [32] and Jiang Zhongzhong [33] proposed TSM models and methods with their own applicability based on different perspectives on different types of TSM problems found in reality. TSM based on preference order has been widely studied by scholars.

TSM can be divided into three types according to the number of individuals involved in the matching results: one-to-one matching, one-to-many matching and many-to-many matching. (1) Under one-to-one (1:1) matching, an individual in Party A can only match with one individual in Party B’s individual set D. For example, in marriage matching, one man can match with only one woman [2, 18]. (2) Under one-to-many (1: n) matching, Party A can match with several individuals in Party B’s individual set D, and an individual from Party B can match with only one individual in Party A's individual set C. In admissions, a school can enroll many students, but a student can choose only one school [3, 19]. (3) Under many-to-many (n: n) matching, Party A can match with many individuals in Party B’s individual set D, and Party B’s individuals may also match with many individuals in Party A’s individual set C. For example, in e-commerce, a seller can have multiple buyers, and a buyer can purchase items from multiple sellers [16].

In this study, on the basis of disappointment theory, we propose a multiobjective optimization model with the objective of maximizing the sum of the perceived utility of hospitals and patients to solve the TSM problem between hospitals and patients. To obtain the preference ranking matrix of the two sides, we rank the matching preference order using the TOPSIS method [36]. Furthermore, we build a model that not only considers two-way selection but also introduces the psychological factors of disappointment theory, which makes the construction of the model more realistic by considering people’s subjective traits rather than completely rational subjects.

## Problem Description and Assumptions

### Notations

The employed mathematical symbols are defined as follows:

${\text{H}} = \left\{ {H_{{1}} , H_{{2}} , \ldots , H_{{\text{i}}} , \ldots , H_{{\text{I}}} } \right\}$: A set of hospitals, where ${\text{H}}_{i}$ is the ith hospital.

$P = \{ P_{1} ,P_{2} , \ldots P_{{\text{j}}} , \ldots P_{{\text{J}}} \}$: A set of patients, where ${\text{P}}_{j}$ is the jth patient.

${\text{H}}_{ - i} = \left\{ {H_{1} ,H_{2} , \ldots H_{i - 1} ,H_{i + 1} , \ldots H_{I} } \right\}$: A set of hospitals without the ith hospital.

${\text{P}}_{{ - {\text{j}}}} = \left\{ {{\text{P}}_{1} ,P_{2} , \ldots P_{{{\text{j}} - 1}} ,P_{{{\text{j}} + 1}} , \ldots P_{{\text{J}}} } \right\}$: A set of patients without the jth patient.

$A = \{ A_{1} ,A_{2} , \ldots A_{{\text{m}}} , \ldots A_{{\text{M}}} \}$: A set of perspectives of hospitals during matching.

$B = \{ B_{1} ,B_{2} , \ldots B_{{\text{n}}} , \ldots B_{{\text{N}}} \}$: A set of perspectives of patients during matching.

$A_{{\text{m}}} = \left\{ {A_{{{\text{m1}}}} ,A_{{{\text{m}}2}} , \ldots A_{{{\text{mg}}}} , \ldots A_{{{\text{mG}}}} } \right\}$: A set of items that influence hospitals’ options under perspective ${\text{A}}_{m}$.

$B_{{\text{n}}} = \left\{ {B_{{{\text{n}}1}} , B_{{{\text{n}}2}} , \ldots B_{{{\text{nk}}}} , \ldots B_{{{\text{nK}}}} } \right\}$: A set of items that influence patients’ options under perspective ${\text{B}}_{n}$.

$R = \left[ {r_{{{\text{ij}}}} } \right]_{I \times J}$: Hospitals’ preference order matrix for patients, where $r_{{{\text{ij}}}}$ denotes that patient P_j_ ranks $r_{{{\text{ij}}}}$ in their preference list of hospitals.

$T = [t_{{{\text{ij}}}} ]_{I \times J}$: Patients’ preference order matrix for hospitals, where ${\mathrm{t}}_{ij}$ denotes that hospital $H_{{\text{i}}}$ ranks $t_{{{\text{ij}}}}$ in their preference list of patients.

${\text{v}}(x)$: Preference utility function.

D(x): The disappointment function.

E(x): The exaltation function.

$V^{{\text{H}}} = \left[ {{\text{v}}\left( {r_{{{\text{ij}}}} } \right)} \right]_{I \times J}$: Hospital preference utility matrix.

$V^{{\text{P}}} = \left[ {{\text{v}}\left( {t_{{{\text{ij}}}} } \right)} \right]_{I \times J}$: Patient preference utility matrix.

$U^{{\text{H}}} = \left[ {{\text{u}}\left( {r_{{{\text{ij}}}} } \right)} \right]_{I \times J}$: Hospitals’ perceived utility matrix.

$U^{{\text{P}}} = \left[ {{\text{u}}\left( {r_{{{\text{ij}}}} } \right)} \right]_{I \times J}$: Patients’ perceived utility matrix.

### Problem Description

In the hospital−patient resource matching system, both hospitals and patients have preference orders for opposing participants according to their evaluations and judgments of each other. Our goal is to provide a stable two-sided satisfied matching proposal for both hospitals and patients that considers the regret perceptions of the two sides.

During matching, we match according to the satisfaction requirements of hospitals and patients and generate two sets of hospitals and patients, which are disjointed: hospital set $H = \{ H_{1} , H_{2} , \ldots , H_{{\text{i}}} , \ldots , H_{{\text{I}}} \}$ and patient set ${\text{P}} = \{ P_{1} ,P_{2} , \ldots P_{{\text{j}}} , \ldots P_{{\text{J}}}$}. We conducted a perception evaluation survey of hospitals and patients. The results show that both sides have different psychological perceptions when matching with different opponents. Therefore, we comprehensively considered the unique preferences of participants' psychological perceptions when establishing the model to provide stable TSM results. The relevant definitions are as follows:

Definition 1 (TSM) [8] Suppose that match Ф involves one-to-one correspondence between the hospitals and patients in a matching system. If hospital ${\text{H}}_{i}$ and patient ${\text{P}}_{j}$ are matched in Ф, then ${\mathrm{H}}_{i}$ and ${\mathrm{P}}_{j}$ are called partners in Ф, and $H_{{\text{i}}}$ = Ф(${\text{P}}_{j}$) and $P_{{\text{j}}}$ = Ф($H_{{\text{i}}}$). ($H_{{\text{i}}}$, $P_{{\text{j}}}$) is called a Ф partner, where Ф($P_{{\text{j}}}$) is the Ф partner of $H_{{\text{i}}}$, and Ф(${\text{H}}_{i}$) is the Ф partner of $P_{{\text{j}}}$.

Definition 2 (Stable TSM) [8] In the case of stable TSM, the preference list of each participant includes all members of the other side. If the hospital and patient match each other and each strictly favors its partner, namely, $H_{i} \succ P_{j} H_{ - i}$ and $P_{j} \succ H_{i} P_{ - j}$, the specific match is stable.

Definition 3 (Degree of satisfaction in TSM) [8] Suppose $\Psi_{ij}$ is the degree of satisfaction of ${\mathrm{H}}_{i}$ with${\mathrm{P}}_{j}$, and ${\Omega }_{ij}$ is the degree of satisfaction of $P_{{\text{j}}}$ with $H_{{\text{i}}}$. Then,

1 $${\Psi }_{ij} = {\text{v}}\left( {r_{ij} } \right), i \in I, j \in J,$$

2 $$\Omega_{ij} = v\left( {t_{ij} } \right), i \in I, j \in J$$

where v(⋅) is a strictly monotonous decreasing function satisfying v(⋅) ≥ 0 and v(1) = 1.

Through field visits and literature research, we found that the indicators affecting how hospitals choose their target patients mainly include four aspects, and five aspects are considered when patients choose hospitals. The details are shown in Tables 1 and 2.

**Table 1:** Evaluation index set of hospitals for patients

Index	Indicator type	Features	Literature sources
Resource urgency	Numerical type (NT)	Cost type (CT)	Chen [20] and Nils [22]
Specialty similarity	Numerical type	Benefit type (BT)	Chen [20] and Wei [21]
Degree of cooperation	Linguistic type (LT)	Benefit type	Varkevisser [23] and Chul [25]
Patience	Linguistic type	Benefit type	Nils [22] and Chul [25]

**Table 2:** Evaluation index set of patients for hospitals

Index	Indicator type	Features	Literature sources
Hospital size (number of beds)	Interval type (IT)	Benefit type	Varkevisser [23], Chul [25], and Smith [26]
Distance to hospital (travel time by car)	Interval type	Cost type	Wei [21], Nils [22], Varkevisser [23], and Liu [24]
Waiting time	Interval type	Cost type	Nils [22] and Varkevisser [23]
Number of parking spaces	Interval type	Benefit type	Smith [26]
Quality of care and service	Interval type	Benefit type	Wei [21], Liu [24], Chul [25], and Smith [26]

### Preference Orders of Hospitals and Patients

TOPSIS (technique for order preference by similarity to the ideal solution) is a commonly used method for comprehensive evaluation based on original data. Its basic principle is to sort by detecting the distance between the evaluation object and the optimal and worst solutions. If the evaluation object is closest to the optimal solution and farthest away from the worst solution, it is the best; otherwise, it is not optimal.

The TOPSIS process is as follows:

Step 1: Index forward. According to different types of indicators, we need to use different formulas for forward processing and thus convert all indicators into very large ones. TOPSIS uses the distance scale to measure the sample gap. If the distance scale is used, the indicator attributes need to be processed in the same direction (if the dataset of one dimension is as large as possible and the dataset of another dimension is as small as possible, scale confusion will result). Cost-based indicators are usually transformed into benefit-based indicators (i.e., the larger the value, higher is the evaluation). Here, all the data become positive.

If the index is cost effective, it will be converted into a benefit index.

If the index is of the interval type, {$\varepsilon_{i}$} is a group of intermediate index series, and the best interval is [a, b]. Then, the positive formula is as follows:

3 $$\varepsilon_{i}^{\prime } = \left\{ {\begin{array}{*{20}c} {1 - \frac{a - \varepsilon }{q},\varepsilon < a} \\ {1, a \le \varepsilon \le b} \\ {1 - \frac{\varepsilon - b}{q},\varepsilon > b} \\ \end{array} } \right. {}q = \max \left\{ {a - \min \left\{ {\varepsilon_{i} } \right\},\max \left\{ {\varepsilon_{i} } \right\} - b} \right\}$$

Step 2: Normalization of the positive matrix. Construct normalized decision matrix $A = \left( {a_{im} } \right)_{I \times M} )$ for the hospitals and matrix $B = (b_{jn} )_{J \times N}$) for the patients in the matching system.

Set ${\text{x}}_{im}$ is the score of hospital i relative to standard m, and ${\text{y}}_{jn}$ is the score of patient j relative to standard n. Normalized values ${\text{a}}_{im}$ and ${\text{b}}_{jn}$ are calculated as follows:

4 $$a_{im} = \frac{{x_{im} }}{{\sqrt {\mathop \sum \nolimits_{i = 1}^{I} \left( {x_{im} } \right)^{2} } }} \quad i = 1,2, \ldots I, m = 1,2 \ldots ,M$$

5 $$b_{jn} = \frac{{y_{jn} }}{{\sqrt {\mathop \sum \nolimits_{j = 1}^{J} \left( {y_{jn} } \right)^{2} } }} \quad j = 1,2, \ldots J, n = 1,2 \ldots ,N$$

Step 3: Construct the weighted normalized decision matrix. Each attribute has its own level of importance, which is represented by a weight. Thus, set of weights {$\omega_{m}$ |m = 1,2,3,…,M} ($\mathop \sum \limits_{m = 1}^{M} \omega_{m}$ = 1) for hospitals’ attributes and set of weights {$\omega_{n}$ |n = 1,2,3,…,N} ($\mathop \sum \limits_{n = 1}^{N} \omega_{n}$ = 1) for patients’ attributes are formulated. The entropy weight method is used to determine the weights in the evaluation index system. Each column of the normalized decision matrix is multiplied by its relevant weight. The elements of new matrices $A\prime = (\omega_{m} a_{im} )_{I \times M}$ and $B\prime = (\omega_{n} b_{jn} )_{J \times N}$ are, respectively, as follows:

6 $$a_{im}^{\prime } = \omega_{m} a_{im} \quad i = 1,2, \ldots ,I, m = 1,2, \ldots ,M$$

7 $$b_{jn}^{\prime } = \omega_{n} b_{jn} \quad j = 1,2, \ldots J, n = 1,2, \ldots ,N.$$

Step 4: Determine the positive ideal and negative ideal solutions (all attributes used in this study are benefit attributes where more attributes is better).

The positive ideal and negative solutions of hospitals are as follows:

8 $$A^{ + } = \left\{ { a_{1 }^{\prime + } , a_{2 }^{\prime + } , \ldots a_{m }^{\prime + } } \right\}$$

where $$a_{m }^{\prime + } = \{ \max \left( {a_{im}^{\prime } } \right)| \, m = 1,2, \ldots ,M\}$$ and

9 $$A^{ - } = \left\{ { a_{1 }^{\prime - } , a_{2 }^{\prime - } , \ldots a_{m }^{\prime - } } \right\}$$

where $$a_{m }^{\prime - } = \{ \min \left( {a_{im} \prime } \right)| \, m = 1,2, \ldots ,M\}$$.

The positive ideal and negative solutions of patients are as follows:

10 $$B^{ + } = \left\{ { b_{1 }^{^{\prime} + } , b_{2 }^{^{\prime} + } , \ldots b_{n }^{^{\prime} + } } \right\}$$

where $$b_{n }^{^{\prime} + } = \{ \max \left( {b_{jn} \prime } \right)| \, n = 1,2, \ldots ,N\}$$ and

11 $$B^{ - } = \left\{ { b_{1 }^{^{\prime} - } , b_{2 }^{^{\prime} - } , \ldots b_{n }^{^{\prime} - } } \right\}$$

where $$b_{n }^{^{\prime} - } = \{ \min \left( {b_{jn} \prime } \right)|n = 1,2, \ldots ,N\}$$.

Step 5: Calculate the distance measurements for each alternative of both sides. For a group of hospitals, the distances from positive ideal and negative ideal choices are as follows:

12 $$C_{i}^{ + } = \sqrt {\mathop \sum \limits_{m = 1}^{M} (a_{m }^{^{\prime} + } - a_{im}^{^{\prime}} )^{2} } {} \quad i = 1,2,3, \ldots ,I$$

13 $$C_{i}^{ - } = \sqrt {\mathop \sum \limits_{m = 1}^{M} (a_{m }^{^{\prime} - } - a_{im}^{^{\prime}} )^{2} } {} \quad i = 1,2,3, \ldots ,I$$

Likewise, the distances from the positive ideal and negative ideal alternatives for a set of patients are, respectively, as follows:

14 $$C_{j}^{ + } = \sqrt {\mathop \sum \limits_{n = 1}^{N} (b_{n }^{^{\prime} + } - b_{jn}^{^{\prime}} )^{2} } {} \quad j = 1,2,3, \ldots ,J$$

15 $$C_{j}^{ - } = \sqrt {\mathop \sum \limits_{n = 1}^{N} (b_{n }^{^{\prime} - } - b_{jn}^{^{\prime}} )^{2} } {} \quad j = 1,2,3, \ldots ,J$$

Step 6: Calculate the relative closeness to the ideal solution of the two sides. Let ${\mathrm{D}}_{i}$ and ${\mathrm{D}}_{j}$ express the general preference utilities of the hospitals and patients, respectively, according to the TOPSIS method.

For a set of hospitals, the following applies:

16 $$D_{i} = \frac{{C_{i}^{ - } }}{{C_{i}^{ + } + C_{i}^{ - } }}$$

For a set of patients, the following applies:

17 $$D_{j} = \frac{{C_{j}^{ - } }}{{C_{j}^{ + } + C_{j}^{ - } }}$$

Step 7: Rank the matching preference orders of the two sides.

According to the comprehensive evaluation value of potential matching objects, the preference ranking matrices of hospitals and patients are obtained as $R = \left[ {r_{ij} } \right]_{I \times J}$ and $T = [t_{ij} ]_{I \times J}$, respectively. In preference ranking matrix $R = \left[ {R_{ij} } \right]_{I \times J}$, $R_{ij}$ represents the preference of hospital ${\text{H}}_{i}$ for patient ${\text{P}}_{j}$. As $R_{ij}$ increases, the hospital’s preference for ${\text{P}}_{j}$ decreases. In particular, when $R_{ij} = { }1$, hospital ${\text{H}}_{i}$ prefers patient ${\text{P}}_{j}$ the most. Similarly, in the patient’s preference ranking matrix ${\text{T }} = [{\text{T}}_{ij} ]_{I \times J}$, ${\text{T}}_{ij}$ represents patient ${\text{P}}_{j}$’s preference for hospital ${\text{H}}_{i}$. As ${\text{T}}_{ij}$ increases, ${\text{P}}_{j}$’s preference for hospital ${\text{H}}_{i}$ decreases. In other words, the preference utility decreases as the preference order increases.

### Perceived Utilities of Hospitals and Patients

In the TSM problem, preference utility refers to the satisfaction of subjects with TSM. To conform to people’s thought patterns, satisfaction can be defined in the range of 0 to 1. A specific explanation is given in definition 3.

Here, ${\text{v}}\left( {r_{ij} } \right)$ denotes hospital ${\text{H}}_{i}$’s preference utility for patient ${\text{P}}_{j}$, and ${\text{v}}\left( {t_{ij} } \right)$ denotes patient${\mathrm{P}}_{j}$’s preference utility for hospital ${\text{H}}_{i}$. ${\text{v}}\left( {r_{ij} } \right)$ and ${\text{v}}\left( {t_{ij} } \right)$ [9] are, respectively, defined by the following formulas:

18 $${\text{v}}\left( {r_{ij} } \right) = \frac{{n + 1 - r_{ij} }}{n}{\text{ i }} \in {\text{ I}},{\text{ j }} \in {\text{ J}}$$

19 $${\text{v}}\left( {t_{ij} } \right) = \frac{{m + 1 - t_{ij} }}{m}{\text{ i }} \in {\text{ I}},{\text{ j }} \in {\text{ J}}$$

$$0 < {\text{v}}\left( {r_{ij} } \right),{\text{v}}\left( {t_{ij} } \right) \le 1$$

**Fig. 1:**
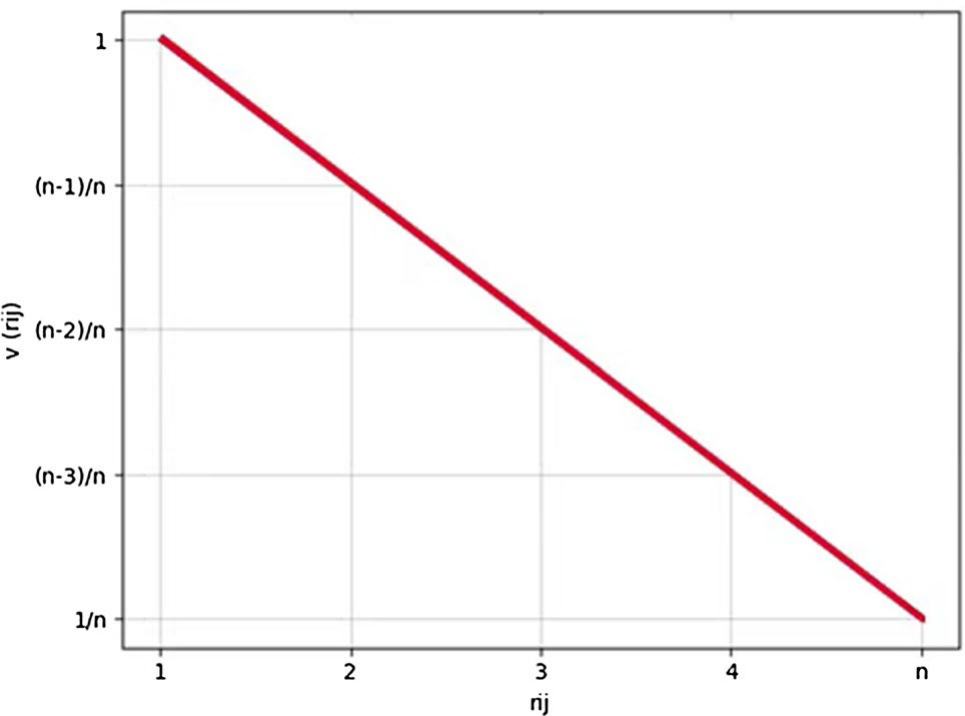
Graphic illustration of the linear function of the degree of satisfaction

Using preference utilities ${\text{v}}\left( {r_{ij} } \right)$ and ${\text{v}}\left( {t_{ij} } \right)$, we are able to build preference utility matrices of hospitals and patients as ${\text{V}}^{H} = \left[ {{\text{v}}\left( {r_{ij} } \right)} \right]_{I \times J}$ and $V^{P} = \left[ {{\text{v}}\left( {t_{ij} } \right)} \right]_{I \times J}$ (i ∈ I, j ∈ J), respectively (Fig. 1).

When considering the psychological and behavioral features of the hospitals and patients, satisfaction with the matching results given by the third party is closely related to the participants’ psychological perceptions of disappointment and elation. In matching result Ф, if hospital ${\text{H}}_{i}$ matches with patient ${\text{P}}_{j}$, the satisfaction of ${\text{H}}_{i}$ with the matching result depends not only on patient ${\text{P}}_{j}$ but also on ${\text{P}}_{ - j}$ because ${\text{H}}_{i}$ will be disappointed if it fails to match with ${\text{P}}_{ - j}$, who is better at matching than ${\text{P}}_{j}$, and it will also be glad if it fails to match with other subjects inferior to ${\text{P}}_{j}$. Similarly, patient ${\text{P}}_{j}$’s satisfaction with matching result Ф depends not only on hospital ${\text{H}}_{i}$ but also on ${\text{H}}_{ - i}$. Based on this, to measure satisfaction with the matching results, we introduce the perceived utility of hospitals and patients with matching results.

According to disappointment theory, ${\text{u}}\left( {r_{ij} } \right)$ denotes hospital ${\text{H}}_{i}$’s psychological perceived [8] utility when matched with ${\text{P}}_{j}$, and ${\text{u}}\left( {t_{ij} } \right)$ denotes patient ${\text{P}}_{j}$’s psychological perceived utility [9] when matched with ${\text{H}}_{i}$. Then,

20 $${\text{u}}\left( {r_{ij} } \right) = \, {\text{v}}\left( {r_{ij} } \right) - \omega_{D} \mathop \sum \limits_{{n:r_{in < } r_{ij} }} \frac{1}{n}D({\text{v}}(r_{in} ) - v\left( {r_{ij} } \right)) + \omega E\mathop \sum \limits_{{n:r_{in > } r_{ij} }} \frac{1}{n}E({\text{v}}(r_{ij} ) - v\left( {r_{in} } \right))$$

21 $${\text{u}}\left( {t_{ij} } \right) =\, {\text{v}}\left( {t_{ij} } \right) - \omega_{D} \mathop \sum \limits_{{m:t_{mj < } t_{ij} }} \frac{1}{m}D({\text{v}}(t_{mj} ) - v\left( {t_{ij} } \right)) + \omega E\mathop \sum \limits_{{m:t_{mj > } t_{ij} }} \frac{1}{m}E({\text{v}}(t_{ij} ) - v\left( {t_{mj} } \right))$$

D(⋅) is the disappointment function, and E(⋅) denotes the excitement function, both of which are nondecreasing functions. ${\text{D}}\left( 0 \right){ } = {\text{ E}}\left( 0 \right){ } = { }0{ }$. ${\upomega }_{{\text{D}}}$ and ${\upomega }_{{\text{E}}}$ are the weights of the disappointment function and excitement function, respectively; ${\upomega }_{{\text{D}}} ,{\upomega }_{{\text{E}}} \ge 0$ and $\omega_{D} + \omega_{E} = 1$.

The disappointment function D(⋅) is as follows:

22 $${\text{D}}\left( x \right) = 1 - \alpha^{x} , x \ge 0$$

**Fig. 2:**
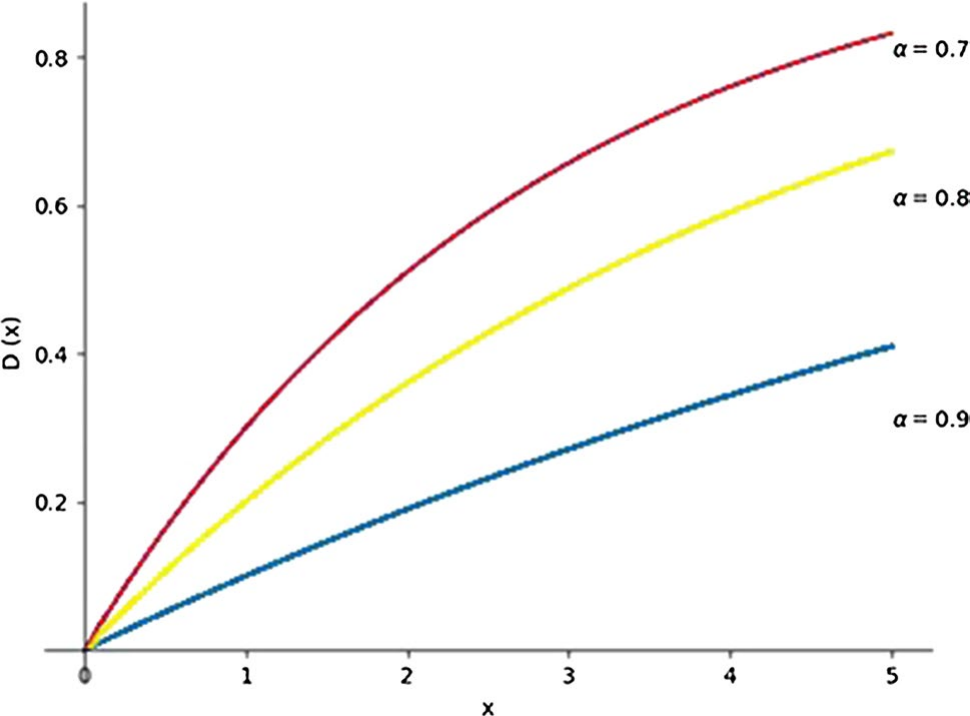
Graphic illustration of the disappointment function

Here, $\mathrm{\alpha }$ is the disappointment parameter, which satisfies $0 < \alpha < 1$; the larger $\mathrm{\alpha }$ is, the lesser the perceived disappointment of the subject is when the matching result is poorer than expected. Laciana and Weber [34] combined the behavioral experimental results of early scholars with a disappointment model and gave the value range of parameter$\mathrm{\alpha }$, which is $0.7 < \alpha < 0.9$. For gap x that fails to meet expectations, the larger $\mathrm{\alpha }$ is, the less disappointed the subject is. A graphic illustration of the disappointment function is shown in Fig. 2.

The elongation function E(x) is as follows:

23 $${\text{E}}\left( x \right) = 1 - \beta^{x} , x \ge 0$$

**Fig. 3:**
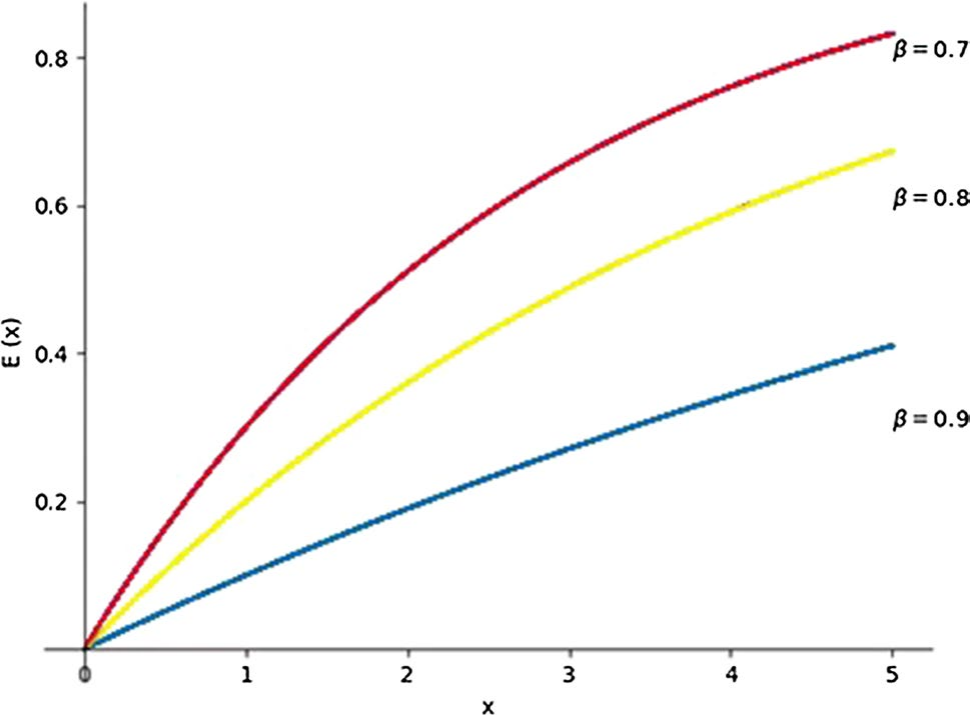
Graphic illustration of the elation function

Here, $\beta$ is the gratification parameter, which satisfies $0 < \beta < 1$; the larger $\beta$ is, the lesser the perceived elation of the subject is when the matching result is higher than expected. The range of parameter $\beta$, which agrees with most of the subjects’ behavioral preferences, is given by Laciana and Weber [34]; namely, $0.7 < \beta < 0.9$. For gap x, the larger $\beta$ is, the less pronounced the subject’s happiness is. A graphic illustration of the elation function is shown in Fig. 3.

Considering the psychological role of hospitals and patients, we use the perceived utility to measure their satisfaction with the matching results. Perceived utility function ${\text{u}}\left( {r_{ij} } \right)$ measures satisfaction with ${\text{H}}_{i}$ and ${\text{P}}_{j}$ matching. The higher ${\text{u}}\left( {r_{ij} } \right)$ is, the greater the satisfaction of ${\text{H}}_{i}$ is. Similarly, ${\text{u}}\left( {t_{ij} } \right)$ measures the satisfaction of ${\text{P}}_{j}$ matching with ${\text{H}}_{i}$. The greater ${\text{u}}\left( {t_{ij} } \right)$ is, the greater the satisfaction of ${\text{P}}_{j}$ is. Under the conditions of ${\text{H}}_{i}$ and ${\text{P}}_{j}$, perceptual utility matrices $U^{{\text{H}}} = \left[ {{\text{u}}\left( {r_{ij} } \right)} \right]_{I \times J}$ and $U^{{\text{P}}} = \left[ {{\text{u}}\left( {r_{ij} } \right)} \right]_{I \times J}$, respectively, are constructed.

## Constructing and Solving the Matching Model

### Construction of a Two‑sided Matching Model

To solve the stable two‑sided matching problem, we introduce 0–1 variables as $x_{ij}$ as follows:

24 $$x_{ij} = \left\{ {\begin{array}{*{20}c} {1, {\text{ if}} \, P_{j} = \left( {H_{i} } \right)} \\ {0 , {\text{if}} \, P_{j} \ne \left( {H_{i} } \right)} \\ \end{array} } \right.$$

Variable ${\mathrm{x}}_{ij}$ indicates that hospital i and patient j are paired as partners; otherwise, they are not matched. For any TSM result, corresponding matching matrix ${\text{X }} = \left[ {x_{ij} } \right]_{I \times J}$ can be constructed.

In addition, a multiobjective optimization model is constructed to maximize the overall perceived utility of the hospitals and patients under the stable TSM constraint and considering the psychological perceptions of the participants.

25a $$\max Z_{{\text{H}}} = \mathop \sum \limits_{i = 1}^{I} \mathop \sum \limits_{j = 1}^{J} x_{ij} {\text{u}}\left( {r_{ij} } \right)$$

25b $$\max Z_{{\text{P}}} = \mathop \sum \limits_{i = 1}^{I} \mathop \sum \limits_{j = 1}^{J} x_{ij} {\text{u}}\left( {t_{ij} } \right)$$

26 $$s.t.\;\;\mathop \sum \limits_{j = 1}^{J} x_{ij} = 1, i \in I$$

27 $$\mathop \sum \limits_{i = 1}^{I} x_{ij} \le 1, j \in J$$

28 $$x_{ij} + \mathop \sum \limits_{{n:r_{in} < r_{ij} }} x_{in} + \mathop \sum \limits_{{m:t_{mj} < t_{ij} }} x_{mj} \ge 1, i \in I,j \in J$$

29 $$x_{ij} \in \left\{ {0,1} \right\} i \in I,j \in J$$

In Model (25), Eqs. (25a) and (25b) are the objective functions, which aim to maximize the sum of hospitals’ perceived utility and patients’ perceived utility as much as possible. Formulas (26) and (27) are the constraint conditions of the 1-to-1 TSM, and Formula (26) is an equality constraint, which means that each hospital must and can only match with one patient. Formula (27) is an inequality constraint requiring that each patient can match with at most one hospital. Formula (28) is a stable TSM constraint, which ensures that the matching result obtained by solving the model is a stable TSM result.

### Solution of the Matching Model

To solve the multiobjective optimization model in the environment of ${\text{I }} \ne {\text{ J}}$, the linear weighting method based on the membership function is used. Let $Z_{H}^{max}$ and ${\text{Z}}_{P}^{max}$ be the optimal values of a single objective and let ${\text{Z}}_{H}^{min}$ and ${\text{Z}}_{P}^{min}$ be the worst values of a single objective, respectively. The membership functions of objective functions (25a) and (25b) are as follows:

30 $$\mu_{{Z_{H} }} = \frac{{Z_{H}^{\max } - Z_{H} }}{{Z_{H}^{\max } - Z_{H}^{\min } }}$$

31 $$\mu_{{Z_{P} }} = \frac{{Z_{P}^{\max } - Z_{P} }}{{Z_{P}^{\max } - Z_{P}^{\min } }}$$

32 $$\min Z = \omega_{H} \mu_{{Z_{H} }} + \omega_{P} \mu_{{Z_{P} }}$$

33 $$s.\,t.\,\mathop \sum \limits_{j = 1}^{J} x_{ij} = 1, i \in I$$

34 $$\mathop \sum \limits_{i = 1}^{I} x_{ij} \le 1, j \in J$$

35 $$x_{ij} + \mathop \sum \limits_{{n:r_{in} < r_{ij} }} x_{in} + \mathop \sum \limits_{{m:t_{mj} < t_{ij} }} x_{mj} \ge 1, i \in I,j \in J$$

36 $$x_{ij} \in \left\{ {0,1} \right\} i \in I,j \in J$$

In the above model, ${\upomega }_{\mathrm{H}}$ and $\omega_{{\text{P}}} \left( {0 \le \omega_{{\text{H}}} ,\omega_{{\text{P}}} \le 1, \omega_{{\text{H}}} + \omega_{{\text{P}}} = 1} \right)$ represent the importance of hospitals and patients for matching decisions, respectively. When ${\upomega }_{H} > {\upomega }_{P}$, more attention will be given to hospital satisfaction in decision making, and when ${\upomega }_{H} < {\upomega }_{P}$, more attention should be given to patient satisfaction in decision making.

## Numerical Examples

In this section, we apply the multiobjective optimization model of TSM to a numerical example. The data for this example were constructed for ease of interpretation.

In the mutual selection process, hospitals usually consider the following four aspects: resource urgency, specialty similarity, the degree of cooperation, and patients. Patients will consider the following five aspects: hospital size (the number of beds), distance to the hospital (travel time by car), wait times, the number of parking spaces, and the quality of care. In the matching system, assume that there are four hospitals H = {H_1_, H_2_, H_3_, H_4_} and five patients P = {P_1_, P_2_, P_3_, P_4_, P_5_}. In the HPM system, each agent has his own preference command among the potential partners of the other party according to their overall evaluation and survey-based judgment. In this matching process, either party strictly selects its partner and no other partner on the opposite side.

Step 1: Extract the preference orders of the hospitals and patients. Hospital preference order matrix ${\text{R }} = { }\left[ {{\text{r}}_{ij} } \right]_{4 \times 5}$ and patient preference order matrix ${\text{T }} = { }[{\text{t}}_{ij} ]_{4 \times 5}$ were developed using the comprehensive evaluation method introduced in the third section.

$$R = \left[ {r_{ij} } \right]_{4 \times 5} = \left[ {\begin{array}{*{20}c} 3 & 4 & {\begin{array}{*{20}c} 5 & 1 & 2 \\ \end{array} } \\ 4 & 3 & {\begin{array}{*{20}c} 1 & 2 & 5 \\ \end{array} } \\ {\begin{array}{*{20}c} 2 \\ 1 \\ \end{array} } & {\begin{array}{*{20}c} 4 \\ 5 \\ \end{array} } & {\begin{array}{*{20}c} {\begin{array}{*{20}c} 3 & 5 & 1 \\ \end{array} } \\ {\begin{array}{*{20}c} 4 & 3 & 2 \\ \end{array} } \\ \end{array} } \\ \end{array} } \right]$$

$$T = [t_{ij} ]_{4 \times 5} = \left[ {\begin{array}{*{20}c} 3 & 1 & {\begin{array}{*{20}c} 2 & 4 & 4 \\ \end{array} } \\ 2 & 2 & {\begin{array}{*{20}c} 1 & 3 & 1 \\ \end{array} } \\ {\begin{array}{*{20}c} 1 \\ 4 \\ \end{array} } & {\begin{array}{*{20}c} 4 \\ 3 \\ \end{array} } & {\begin{array}{*{20}c} {\begin{array}{*{20}c} 3 & 2 & 3 \\ \end{array} } \\ {\begin{array}{*{20}c} 4 & 1 & 2 \\ \end{array} } \\ \end{array} } \\ \end{array} } \right]$$

Step 2: Calculate the preference utilities of hospitals and patients.

According to the Formulas (18) and (19), preference order value matrices R and T are transformed into preference utility matrices ${\text{V}}^{H} = [{\text{v}}({\text{r}}_{ij} )]_{4 \times 5}$ and ${\text{V}}^{P} = [{\text{v}}({\text{t}}_{ij} )]_{4 \times 5}$, respectively:

$${\text{V}}^{H} = [{\text{v}}({\text{r}}_{ij} )]_{4 \times 5} = \left[ {\begin{array}{*{20}c} {0.60} & {0.40} & {\begin{array}{*{20}c} {0.20} & {1.00} & {0.80} \\ \end{array} } \\ {0.40} & {0.60} & {\begin{array}{*{20}c} {1.00} & {0.80} & {0.20} \\ \end{array} } \\ {\begin{array}{*{20}c} {0.80} \\ {1.00} \\ \end{array} } & {\begin{array}{*{20}c} {0.40} \\ {0.20} \\ \end{array} } & {\begin{array}{*{20}c} {\begin{array}{*{20}c} {0.60} & {0.20} & {1.00} \\ \end{array} } \\ {\begin{array}{*{20}c} {0.40} & {0.60} & {0.80} \\ \end{array} } \\ \end{array} } \\ \end{array} } \right]$$

$${\text{V}}^{P} = [{\text{v}}({\text{t}}_{ij} )]_{4 \times 5} = \left[ {\begin{array}{*{20}c} {0.50} & {1.00} & {\begin{array}{*{20}c} {0.75} & {0.25} & {0.25} \\ \end{array} } \\ {0.75} & {0.75} & {\begin{array}{*{20}c} {1.00} & {0.50} & {1.00} \\ \end{array} } \\ {\begin{array}{*{20}c} {1.00} \\ {0.25} \\ \end{array} } & {\begin{array}{*{20}c} {0.25} \\ {0.50} \\ \end{array} } & {\begin{array}{*{20}c} {\begin{array}{*{20}c} {0.50} & {0.75} & {0.50} \\ \end{array} } \\ {\begin{array}{*{20}c} {0.25} & {1.00} & {0.75} \\ \end{array} } \\ \end{array} } \\ \end{array} } \right]$$

Step 3: Calculate the perceived utilities of hospitals and patients.

The range of disappointment parameters $\mathrm{\alpha }$ and $\upbeta$ is $0.7 \le \alpha , \beta \le 0.9$. Here, assuming that the degree of disappointment avoidance of both parties is moderate, $\mathrm{\alpha }$=$\upbeta$=0.8. In addition, we believe that the disappointment and elation of hospitals and patients are equally important, so $\omega_{D} = \,\omega_{E} \, = 0.5$. Perceived utility matrices ${\text{U}}^{H} = [{\text{u}}({\text{r}}_{ij} )]_{4 \times 5}$ and ${\text{U}}^{P} = [{\text{u}}({\text{t}}_{ij} )]_{4 \times 5}$ are thus constructed:

$${\text{U}}^{H} = [{\text{u}}({\text{r}}_{ij} )]_{4 \times 5} = \left[ {\begin{array}{*{20}c} {0.60} & {0.38} & {\begin{array}{*{20}c} {0.16} & {1.04} & {0.82} \\ \end{array} } \\ {0.38} & {0.60} & {\begin{array}{*{20}c} {1.04} & {0.82} & {0.16} \\ \end{array} } \\ {\begin{array}{*{20}c} {0.82} \\ {1.04} \\ \end{array} } & {\begin{array}{*{20}c} {0.38} \\ {0.16} \\ \end{array} } & {\begin{array}{*{20}c} {\begin{array}{*{20}c} {0.60} & {0.16} & {1.04} \\ \end{array} } \\ {\begin{array}{*{20}c} {0.38} & {0.60} & {0.82} \\ \end{array} } \\ \end{array} } \\ \end{array} } \right]$$

$${\text{U}}^{P} = [{\text{u}}({\text{t}}_{ij} )]_{4 \times 5} = \left[ {\begin{array}{*{20}c} {0.49} & {1.04} & {\begin{array}{*{20}c} {0.76} & {0.21} & {0.21} \\ \end{array} } \\ {0.76} & {0.76} & {\begin{array}{*{20}c} {1.04} & {0.49} & {1.04} \\ \end{array} } \\ {\begin{array}{*{20}c} {1.04} \\ {0.21} \\ \end{array} } & {\begin{array}{*{20}c} {0.21} \\ {0.49} \\ \end{array} } & {\begin{array}{*{20}c} {\begin{array}{*{20}c} {0.49} & {0.76} & {0.49} \\ \end{array} } \\ {\begin{array}{*{20}c} {0.21} & {1.04} & {0.76} \\ \end{array} } \\ \end{array} } \\ \end{array} } \right]$$

Step 4: Construct the TSM model.

A multiobjective optimization model was constructed to maximize the overall perceived utility of both hospitals and patients:

37 $$\max Z_{H} = \mathop \sum \limits_{i = 1}^{4} \mathop \sum \limits_{j = 1}^{5} x_{ij} u\left( {r_{ij} } \right)$$

38 $$\max Z_{P} = \mathop \sum \limits_{i = 1}^{4} \mathop \sum \limits_{j = 1}^{5} x_{ij} u\left( {t_{ij} } \right)$$

39 $${\text{s}}.\,{\text{t}}. \to \,\;\mathop \sum \limits_{j = 1}^{5} x_{ij} = 1, i \in \left\{ {1,2,3,4} \right\}$$

40 $$\mathop \sum \limits_{i = 1}^{4} x_{ij} \le 1, j \in \left\{ {1,2,3,4,5} \right\}$$

41 $$x_{ij} + \mathop \sum \limits_{{n:r_{in} < r_{ij} }} x_{in} + \mathop \sum \limits_{{m:t_{mj} < t_{ij} }} x_{mj} \ge 1, i \in \left\{ {1,2,3,4} \right\},j \in \left\{ {1,2,3,4,5} \right\}$$

42 $$x_{ij} \in \left\{ {0,1} \right\} i \in \left\{ {1,2,3,4} \right\},j \in \left\{ {1,2,3,4,5} \right\}$$

Step 5: Solve the TSM model.

According to perceived utility matrices U^H^ and U^P^, upon using the LINGO 11.0 software package to solve the multiobjective optimization model, it can be concluded that $Z_{H}^{\max } = 2.84$, $Z_{H}^{\min } = 1.96,$ $Z_{P}^{\max } = 4.16,\,\,and \,Z_{P}^{\min } = 1.39$ When considering the fairness of both hospitals and patients, hospitals and patients are equally important; hence, $\omega_{H} = \omega_{P} = 0.5$. The weighted sum method based on the membership function is used to construct the following single objective programming model:

43 $$\min Z = 2.36 - \mathop \sum \limits_{i = 1}^{4} \mathop \sum \limits_{j = 1}^{5} \left[ {0.57u\left( {r_{ij} } \right) + 0.18u\left( {t_{ij} } \right)} \right]x_{ij}$$

44 $${\text{s}}.\,{\text{t}}.\,\;\mathop \sum \limits_{j = 1}^{5} x_{ij} = 1, i \in \left\{ {1,2,3,4} \right\}$$

45 $$\mathop \sum \limits_{i = 1}^{4} x_{ij} \le 1, j \in \left\{ {1,2,3,4,5} \right\}$$

46 $$x_{ij} + \mathop \sum \limits_{{n:r_{in} < r_{ij} }} x_{in} + \mathop \sum \limits_{{m:t_{mj} < t_{ij} }} x_{mj} \ge 1, i \in \left\{ {1,2,3,4} \right\},j \in \left\{ {1,2,3,4,5} \right\}$$

47 $$x_{ij} \in \left\{ {0,1} \right\} i \in \left\{ {1,2,3,4} \right\},j \in \left\{ {1,2,3,4,5} \right\}$$

The optimal solution and the objective value obtained by LINGO 11.0 are shown as follows:

$$X^{*} = \left( {x_{ij}^{*} } \right)_{4 \times 5} = \left[ {\begin{array}{*{20}c} 0 & 0 & {\begin{array}{*{20}c} 1 & 0 & 0 \\ \end{array} } \\ 0 & 0 & {\begin{array}{*{20}c} 0 & 0 & 1 \\ \end{array} } \\ {\begin{array}{*{20}c} 0 \\ 0 \\ \end{array} } & {\begin{array}{*{20}c} 0 \\ 1 \\ \end{array} } & {\begin{array}{*{20}c} {\begin{array}{*{20}c} 0 & 1 & 0 \\ \end{array} } \\ {\begin{array}{*{20}c} 0 & 0 & 0 \\ \end{array} } \\ \end{array} } \\ \end{array} } \right]$$, Z* = 0.92

The solution of two-sided satisfied stable matching is Ф^∗^ = {(1, 3), (2, 5), (3, 4), (4, 2)}. The results indicate that hospital H_1_ and patient P_3_ match, hospital H_2_ and patient P_5_ match, hospital H_3_ and patient P_4_ match, hospital H_4_ and patient P_2_ match and P_1_ is left unmatched.

To further illustrate the practical significance of considering disappointment theory in solving the two-sided satisfactory matching problem, the following analysis is given.

In this example, if the psychological perception of matching is not considered, then ${\text{u}}\left( {t_{ij} } \right) = {\text{v}}\left( {t_{ij} } \right),{\text{u}}\left( {r_{ij} } \right) = {\text{v}}\left( {r_{ij} } \right)$, and the matching matrix can be obtained by solving the model:

$$X^{\prime} = \left( {x_{ij}^{^{\prime}} } \right)_{4 \times 5} = \left[ {\begin{array}{*{20}c} 1 & 0 & {\begin{array}{*{20}c} 0 & 0 & 0 \\ \end{array} } \\ 0 & 0 & {\begin{array}{*{20}c} 0 & 0 & 1 \\ \end{array} } \\ {\begin{array}{*{20}c} 0 \\ 0 \\ \end{array} } & {\begin{array}{*{20}c} 0 \\ 1 \\ \end{array} } & {\begin{array}{*{20}c} {\begin{array}{*{20}c} 0 & 1 & 0 \\ \end{array} } \\ {\begin{array}{*{20}c} 0 & 0 & 0 \\ \end{array} } \\ \end{array} } \\ \end{array} } \right]$$, Z* = 0.78.

The two-sided satisfied stable matching result is Ф^∗^ = {(1, 1), (2, 5), (3, 4), (4, 2)}.

Upon comparing the two matching results, we can see that overall satisfaction with match $X^{\prime}$ is lower than that of $X^{*}$. If the matching results of hospital patients are determined according to $X^{\prime}$, patient $P_{3}$ will be dissatisfied because he wants to go to $H_{1}$ more than $P_{1}$ but is not selected, and the hospital will also be dissatisfied because it is more willing to receive patient $P_{3}$. Therefore, choosing match $X^{*}$ as the optimal matching result has better practical significance in this case.

## Conclusion

In this paper, we consider the overall satisfaction and fairness weights of both hospitals and patients, which can maximize the sum of the perceived utility of both actors. The results show that both hospitals and patients can effectively solve the uncertainty problem of stable TSM by transforming preferred utility into perceived utility. Through a comparison of the multiobjective optimization model of TSM with disappointment theory and the model without disappointment theory, we find that the results considering psychological factors can better determine the overall satisfaction of hospitals and patients. It is concluded that this method can help local health management institutions meet the needs of hospitalized patients to the greatest extent through the rational allocation of limited medical resources to alleviate the contradiction between the supply and demand of medical resources. Therefore, we recommend that the MRI consider the psychological behavior and subjective views of both sides when managing hospital patient resource matching rather than judging from a perspective of complete rationality.

One limitation of this study is that when considering the preference order of both parties, this paper presents the score of the hospital and patient evaluation index based on the TOPSIS method, but it is difficult to select a quantitative index corresponding to the data of each index. In addition, due to the large scale of real TSM problems, it is unrealistic to build and solve such an optimization model manually, and thus determining how to embed the matching algorithm into a decision support system to enhance practicability and operability could be a very interesting topic. We anxiously await future extensions of the present study.

## Data Availability

The data involved in this paper is mainly used for simulation experiments, not real data.

## Abbreviations

TOPSIS: Technique for order preference by similarity to an ideal solution
MRI: Medical research institute
TSM: Two-Sided matching
HPM: Hospital−patient matching
CT: Cost type
BT: Benefit type
IT: Interval type
LT: Linguistic type
NT: Numerical type
